# The Eye and Its Care

**Published:** 1897-03

**Authors:** 


THE EYE AND ITS CARE. By FRANK ALLPORT, M. D., Minneapolis, Minn., Professor of Clinical Ophthalmology and Otology in the Minnesota State University; President of the Minnesota State Medical Society; Secretary of the Ophthalmological Section of the American Medical Association, etc. Illustrated. 12mo, pp. 174. Philadelphia. J. B. Lippincott Co. 1896.

This little work is designed for popular instruction. It takes up in a general and nontechnical way the anatomy and physiology of the eye, refraction, glasses, and the care of the eyes. It does not, like many of its class, undertake to become a treatise on diseases of the eye, but it intelligibly presents just such information as laymen ought to possess, especially teachers. A. A. H.

